# Low level bacterial endotoxin activates two distinct signaling pathways in human peripheral blood mononuclear cells

**DOI:** 10.1186/1476-9255-8-4

**Published:** 2011-02-25

**Authors:** Andra L Blomkalns, Lynn L Stoll, Wassim Shaheen, Sara A Romig-Martin, Eric W Dickson, Neal L Weintraub, Gerene M Denning

**Affiliations:** 1 Department of Emergency Medicine, University of Cincinnati, Cincinnati, OH USA; 2 Department of Emergency Medicine, Roy J. And Lucille A. Carver College of Medicine, University of Iowa, Iowa City, IA, USA; 3 The Department of Internal Medicine, Division of Cardiovascular Diseases, Roy J. And Lucille A. Carver College of Medicine, University of Iowa, Iowa City, IA, USA; 4 Department of Emergency Medicine, University of Massachusetts Medical School, Worcester, MA, USA; 5 Department of Internal Medicine, Division of Cardiovascular Diseases, University of Cincinnati, Cincinnati, OH, USA

## Abstract

**Background:**

Bacterial endotoxin, long recognized as a potent pro-inflammatory mediator in acute infectious processes, has more recently been identified as a risk factor for atherosclerosis and other cardiovascular diseases. When endotoxin enters the bloodstream, one of the first cells activated is the circulating monocyte, which exhibits a wide range of pro-inflammatory responses.

**Methods:**

We studied the effect of low doses of *E. coli *LPS on IL-8 release and superoxide formation by freshly isolated human peripheral blood mononuclear cells (PBMC).

**Results:**

IL-8 release was consistently detectable at 10 pg/ml of endotoxin, reaching a maximum at 1 ng/ml, and was exclusively produced by monocytes; the lymphocytes neither produced IL-8, nor affected monocyte IL-8 release. Superoxide production was detectable at 30 pg/ml of endotoxin, reaching a maximum at 3 ng/ml. Peak respiratory burst activity was seen at 15-20 min, and superoxide levels returned to baseline by 1 h. IL-8 release was dependent on both membrane-associated CD14 (mCD14) and Toll-like receptor 4 (TLR4. Superoxide production was dependent on the presence of LBP, but was not significantly affected by a blocking antibody to TLR4. Moreover, treatment with lovastatin inhibited LPS-dependent IL-8 release and superoxide production.

**Conclusions:**

These findings suggest that IL-8 release and the respiratory burst are regulated by distinct endotoxin-dependent signaling pathways in PBMC in low level of endotoxin exposure. Selectively modulating these pathways could lead to new approaches to treat chronic inflammatory diseases, such as atherosclerosis, while preserving the capacity of monocytes to respond to acute bacterial infections.

## Background

Bacterial endotoxin, long recognized as a potent pro-inflammatory mediator in acute infectious processes, has more recently been identified as a risk factor for atherosclerosis and other forms of cardiovascular disease [1-3]. In endotoxemia, one of the first cells to elicit a pro-inflammatory response is the circulating monocyte. Monocytes are known to respond to extremely low levels of endotoxin (pg/ml) [3], producing a wide array of chemokines and cytokines, and releasing cytotoxic levels of reactive oxygen species (ROS) *via *the respiratory burst. The resulting inflammatory process is initially beneficial to the host as a means of protection against invading microorganisms; however, if not resolved, chronic damage to host tissues can ensue. In this respect, a growing body of evidence suggests that the persistent low-level inflammation associated with chronic, subclinical infections (*e.g*., periodontitis, diverticulitis, or smoker's bronchitis) may also play a role in exacerbation of vascular diseases such as atherosclerosis [1].

The classic endotoxin signaling pathway has been shown to involve LBP, CD14, MD-2, and Toll-like receptor 4 (TLR4)[3-6]. It was commonly believed that all of these elements are required for initiation of endotoxin signaling[7], Although this pathway has been extensively documented in many model systems, there is evidence that alternate signaling pathways may exist in some cells. For example, endotoxin concentrations >100 ng/ml have been shown to activate host cells by mechanisms independent of the CD14-TLR4 pathway [8-10] Moreover, rapid production of ROS in LPS-simulated (100 ng/mL) macrophages has been shown to be partly dependent on the activation of cytoplasmic GTPase Rac1, a known activator of NOX-1 oxidase enzyme activity[11]. The above findings may be pertinent to mechanisms of LPS signaling in the setting of acute sepsis. In contrast, relatively little is known about potential alternative signaling pathways in humans activated by lower levels of endotoxin pertinent to chronic inflammatory diseases such as atherosclerosis.

To address this question, we designed experiments using freshly isolated human peripheral blood mononuclear cells (PBMC) to compare and contrast the effects of low levels of endotoxin (≤1 ng/ml) on two inflammatory responses, IL-8 release and superoxide production. Our data suggest that two distinct signaling pathways are involved in eliciting these responses. The presence of these two pathways in monocytes raises the possibility of targeting therapeutic interventions to modulate specific pro-inflammatory responses involved in chronic inflammatory diseases such as atherosclerosis.

## Methods

### Materials

The endotoxin (LPS) from E. coli K12 LCD25 was purchased from List Biological Laboratories. All LPS preparations from List Biological Laboratories, Inc., are essentially free of nucleic acid and protein and are chemically characterized with respect to their phosphate and 2-keto-3-deoxyoctonate (KDO) contents. For these studies, the purchased LPS preparation was further purified by a phenol re-extraction method to eliminate residual protein contamination prior to use in our experimental models.

MEM-18, an antibody that binds to the endotoxin-binding site of CD14, was purchased from Accurate Chemical (Westbury, NY). Blocking antibodies to TLR4 (HTA-125) and to CD11, CD11b, and CD18 were purchased from eBioscience (San Diego, CA). Recombinant LBP was purchased from R&D Systems (Minneapolis, MN). Lucigenin (bis-N-methylacridinium nitrate), and lovastatin were purchased from Sigma Aldrich (St. Louis, MO). Human serum albumin (HSA) was obtained from the University of Iowa Hospital pharmacy. Lipooligosaccharide (LOS) from *N. meningitides *was generously provided by Dr. Michael Apicella (University of Iowa).

### PBMC isolation

Heparinized venous blood was obtained from healthy volunteers (n = 4) in accordance with a protocol approved by the Institutional Review Board for Human Subjects at the University of Iowa. Peripheral blood mononuclear cells (PBMC) were isolated using dextran sedimentation and Hypaque-Ficoll density-gradient separation followed by hypotonic lysis of erythrocytes as previously described [12]. Generally monocytes comprise approximately 10% of the isolated PBMCs. Purified PBMC were resuspended in incubation buffer (sterile pyrogen-free Hanks balanced salt solution [HBSS; Bio-Whittaker] supplemented with 0.1% D-glucose [HBSS/G] and 0.1% human serum albumin [HSA]) and kept on ice until use.

### IL-8 release

For experiments using whole PBMC, cells were suspended at 1 × 10^7 ^PBMC/ml in incubation buffer with 2× the indicated final concentration of LBP. Aliquots (100 μl) of cell suspension were then placed in individual wells of a 48-well tissue culture plate, and 100 μl of 2× the indicated final concentration of endotoxin was added to the wells.

For studies to determine the relative contribution of the monocyte and lymphocyte subpopulations, 1 × 10^6 ^PBMC in 100 μl of HBSS/G were placed in wells of a 48-well plate, and cultures were incubated at 37°C for 2 h to allow the monocytes to adhere. The buffer containing the non-adherent cells (primarily lymphocytes) was then removed and pooled. The cells were pelleted and resuspended in incubation buffer (1 × 10^7^/ml) with 2× the indicated concentration of LBP. 100 μl of cell suspension was placed in individual wells of a 48-well plate, and 100 μl of 2× the indicated final concentration of endotoxin was then added to the wells. The adherent cells were gently washed twice with HBSS at *37°C *containing 0.1% HSA; 200 μl of incubation buffer containing indicated concentrations of endotoxin and LBP was then added to each well. For all studies, cultures were incubated in a 37°C for 6 h. At the end of this incubation period, the media were transferred to microfuge tubes and centrifuged for 2 min at 5,000 rpm. Supernatants from these samples were then transferred to new microfuge tubes and frozen until analysis. IL-8 was measured by ELISA, using matched antibodies from R&D Systems as described previously. The lower limit of detection for this IL-8 assay was 15 pg/ml[13].

### Lucigenin-enhanced chemiluminescence

Lucigenin-enhanced chemiluminescence was measured by a modification of the method of Allen *et al*. [14] using a microplate luminometer (FLUOStar Optima, BMG Labtech, Durham, NC). All reagents were prepared using sterile pyrogen-free injectable solutions obtained from the hospital pharmacy, and all components of the reaction were combined on ice to prevent premature initiation of the respiratory burst. Isolated PBMC were diluted to a final concentration of 1.0 × 10^7^/ml in ice-cold incubation buffer with 2× the indicated final concentration of LBP, and 100 μl aliquots of the cell suspension were placed in wells of a 96-well Optiplate (Perkin Elmer, Boston, MA). Lucigenin (final concentration of 1 × 10^-4 ^M) and 2× the indicated final concentration of endotoxin were then added to each well. The plates were then placed in the luminometer and two-sec readings were taken at 37°C every 2 min for 180 min. The resulting readings in relative light units (LU) were summed to provide a measure of activity. Values are expressed as the sum of the readings in arbitrary light units (LU) collected over the 2 h measurement, and represent the mean + SEM for 4 replicate samples.

### Inhibitor and blocking antibody studies

To determine the effect of lovastatin, cells were pre-incubated overnight at 37°C with 1.5 μM of the drug in incubation buffer, and then stimulated with the indicated concentration of endotoxin. To test the effect of blocking antibodies, 1 × 10^6 ^PBMC in 100 μl of incubation buffer with the indicated concentration of LBP and with the indicated antibody were incubated for 30 min at 37°C prior to endotoxin addition. Antibodies tested were: 1 μg/well MEM18, anti-CD14 (Leinco Technologies), and 2 μg/well HTA-125, anti-TLR4.

### Statistics

All statistical analyses were performed with SigmaStat version 3.1 (SPSS Inc., Chicago, IL). Data are expressed as means ± SEM and have been compared by Student's t-test and repeated measures ANOVA when appropriate. *P < 0.05 was considered to be statistically significant.

## Results

### Effect of endotoxin on IL-8 release by human PBMC

**Figure 1:**
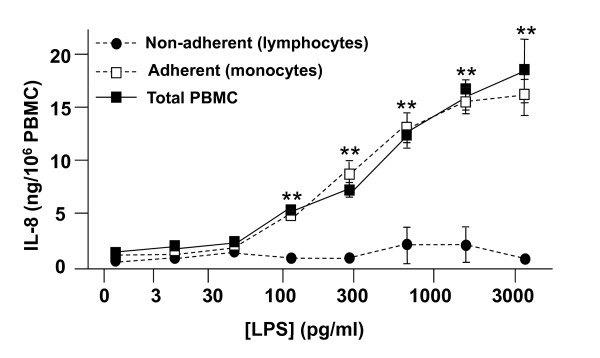
**Effect of LPS on IL-8 release by adherent (monocytes) and non-adherent (lymphocytes) cells**. Freshly isolated human PBMC (~1.0 × 10^6 ^total cells/well, ~100,000 monocytes), as well as adherent and non-adherent cell fractions from the same donor, were prepared as described in **Methods **in 200 μl of incubation buffer, 100 ng/ml of LBP, and the indicated concentration of endotoxin. Cultures were then incubated at 37°C for 6 h. At the end of the incubation period, cells were pelleted, and buffer samples were frozen at -20°C for subsequent IL-8 analysis by ELISA. Data are expressed as the mean ± S.E.M. of triplicate samples; error bars for some conditions were so small they are obscured by the symbols. **p < 0.001 for total PBMC and non-adherent cells relative to the no LPS control, and in comparison with adherent cells. Similar results were seen in 3 independent experiments and in a parallel series of experiments with *N. meningitidis *LOS (data not shown).

Figure 1 demonstrates that LPS concentrations as low as 3 pg/mL resulted in measurable IL-8 production (2.10 ng of IL-8 per million total PBMCs) from freshly isolated human PBMCs (squares) under the tested conditions. The response is significant at LPS concentrations of 100 pg/mL, achieving a maximal level at ~1 ng/mL. This figure also shows that the IL-8 release is due to adherent cells (monocytes, diamonds). Non-adherent cells (primarily lymphocytes, circles) produced little or no measurable IL-8 over the concentration range tested, nor did their presence appear to affect monocyte IL-8 release. Similar results were obtained in experiments using *N. meningitidis *lipooligosaccharide (LOS; data not shown).

**Figure 2:**
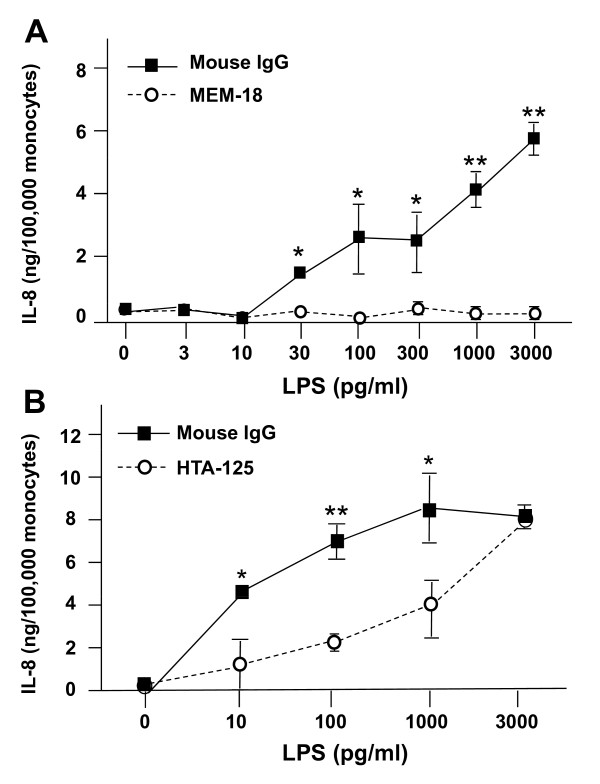
**Effect of blocking antibodies on endotoxin-dependent IL-8 release by PBMC**. Freshly isolated human PBMC (~1.0 × 10^6 ^total cells/well, 100,000 monocytes) were prepared as described in **Methods **in 100 μl of incubation buffer containing 100 ng/ml LBP, and (A) 1 μg/well of non-immune mouse IgG or MEM-18 (anti-CD14), or (B) 2 μg/well non-immune mouse IgG or HTA-125 (anti-TLR4). Cultures were then pre-incubated at 37°C for 30 min, and 100 μl of 2× the final endotoxin concentration in incubation buffer was added to the wells. Cultures were incubated at 37°C for 6 h. At the end of the incubation period, samples were collected and frozen for subsequent IL-8 analysis by ELISA. Data are expressed as the mean ± S.E.M. of triplicate samples; error bars for some conditions were so small they are obscured by the symbols. (A) *p < 0.05, **p < 0.001 for mouse IgG values relative to MEM-18 values for the same LPS concentration. (B) *p < 0.05, **p < 0.001 for mouse IgG values relative to HTA-125 values for the same LPS concentration. Similar results were seen in 3 independent experiments.

Endotoxin signaling reportedly requires CD14 (membrane or soluble), MD-2, and TLR4 [4,15,16]. To determine whether these proteins are involved in LPS-induced IL-8 release by human PBMC, we used blocking antibodies. Figure 2 shows that antibodies to CD14 (MEM-18; Figure 2A) and to TLR4 (HTA-125; Figure 2B) inhibited LPS-induced IL-8 release; however, the HTA-125 antibody was less effective at higher LPS concentrations. To confirm the specificity of these antibodies, we performed parallel experiments with TNFα as a stimulus. Neither MEM-18 nor HTA-125 inhibited TNFα-stimulated IL-8 release (data not shown).

### Effect of increasing LBP on endotoxin-dependent IL-8 release by monocytes and macrophages

**Figure 3:**
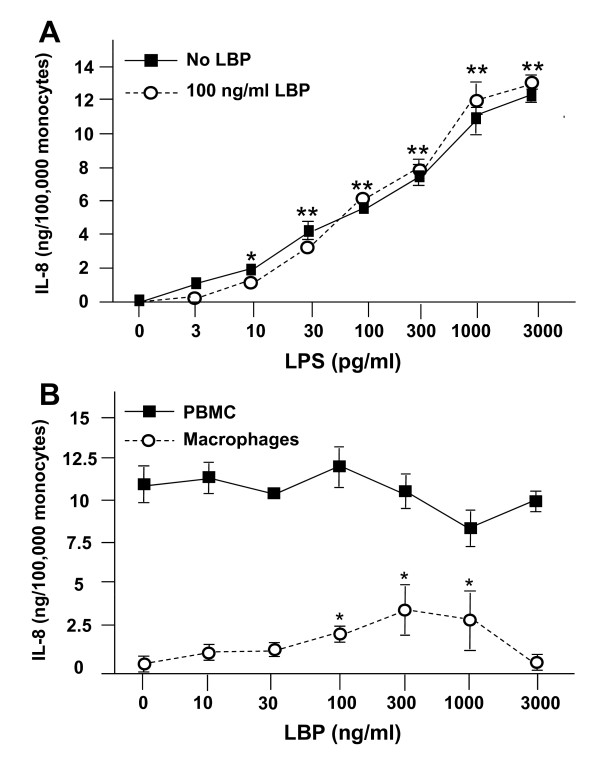
**Effect of LBP on endotoxin-dependent IL-8 release by PBMC**. (A) Freshly isolated human PBMC (~1.0 × 10^6 ^total cells/well, 100,000 monocytes) were prepared as described in **Methods **in 200 μl of incubation buffer with and without 100 ng/ml LBP, and with the indicated concentration of endotoxin. (B) PBMC and partially differentiated macrophages (~100,000 cells/well; see **Methods**) from the same donor were prepared in 200 μl of the same buffer containing 100 pg/ml of endotoxin and the indicated concentration of LBP. All cultures were then incubated for 6 h. At the end of the incubation period, samples were collected and frozen for subsequent IL-8 analysis by ELISA. Data are expressed as the mean ± S.E.M. of triplicate samples; error bars for some conditions were so small they are obscured by the symbols. (A) *p < 0.05, **p < 0.001 relative to the no LPS control. There were no significant differences between the presence and absence of LBP. (B) *p < 0.05 relative to the no LBP control. Similar results were seen in 3 independent experiments.

LBP is a 60 kDa lipid/phospholipid binding and transfer protein with fairly broad specificity [17,18]. Its role with respect to endotoxin is to catalyze the delivery and transfer of monomeric endotoxin to either membrane-bound (mCD14) or soluble CD14 (sCD14) for cellular activation. Plasma LBP levels have been shown to increase by 10- to 30-fold during acute inflammatory responses [19]. Physiological levels of LBP facilitate inflammation, while the high levels of LBP in acute inflammatory responses are inhibitory [20]. Therefore, we tested the effect of increasing LBP concentrations on LPS-induced IL-8 release by PBMC (Figure 3). Surprisingly, LPS-induced IL-8 release by PBMCs did not change over the range of LPS concentrations tested (Figure 3A).

The lipid A moiety of endotoxin requires protein binding for resultant cellular activation. We hypothesized that an endotoxin-binding protein other than LBP was involved in this response. Studies by *Gioannini et al*. [21] have found that albumin is an essential and specific facilitator of LBP/sCD14-induced LOS disaggregation. To determine whether albumin alone present in our medium was sufficient for monocytic cellular activation, we tested the effect of decreasing albumin concentrations (from 100 nM to 0 nM) on IL-8 release. We saw decreasing IL-8 release with decreasing albumin concentrations (data not shown), suggesting an albumin dependent reaction and is consistent with the known interactions of albumin and endotoxin.

Since macrophages have been reported to exhibit an LBP-dependent inflammatory response [18], we next compared the effect of increasing LBP on LPS-induced IL-8 release by freshly isolated PBMC, and by 3-day differentiated macrophages from the same donor (Figure 3B). As previously reported, there was an LBP-dependent increase in IL-8 release by differentiated macrophages (Figure 3B, circles) that was followed by a decrease at higher LBP concentrations (3 ng/ml); macrophages produced significantly less IL-8 relative to PBMC at all LBP concentrations tested. In contrast, increasing LBP had little or no effect on LPS-mediated IL-8 release by freshly isolated monocytes from the same donor (Figure 3B, squares). Similar findings were observed in studies using *N. meningitidis *LOS (data not shown).

### Effect of endotoxin on the respiratory burst in PBMC

**Figure 4:**
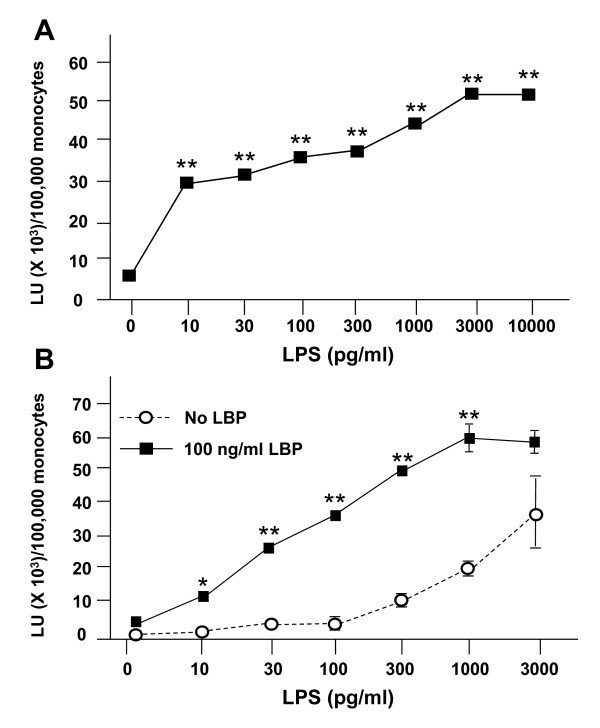
**Effect of LBP on endotoxin-induced superoxide production by PBMC**. (A) Freshly isolated human PBMC (~1.0 × 10^6 ^total cells/well, 100,000 monocytes) were placed on ice in individual wells of a 96-well white Optiplate in a total volume of 200 μl of incubation buffer containing 0.1 mM lucigenin, 100 ng/ml of LBP, and the indicated concentration of endotoxin. (B) PBMC were prepared under the same conditions with and without 100 ng/ml LBP and the indicated concentration of endotoxin. The plates were then placed in the luminometer and two-sec readings were taken at 37°C every 2 min for 180 min. The resulting readings in relative light units (LU) were summed to provide a measure of activity. Data are expressed as total LU (in thousands) per 100,000 monocytes and represent the mean ± S.E.M. of 4 replicate samples; error bars for some conditions were so small they are obscured by the symbols. (A) **p < 0.001 relative to the no LPS control. (B) *p < 0.05, **p < 0.001 for the 100 ng/ml LBP values relative to the no LBP values at the same LPS concentration. Similar results were seen in 3 independent experiments, and in a parallel series of experiments with *N. meningitidis *LOS (not shown).

As part of the innate immune response to pathogens, monocytes reduce molecular oxygen to superoxide anion, a process known as the respiratory burst. To determine the effect of LPS on the respiratory burst in PBMC, we used lucigenin-enhanced chemiluminescence. Figure 4A demonstrates that LPS caused a concentration-dependent increase in superoxide production. As with IL-8 release (Figure 1), superoxide release by PBMC is due primarily to the adherent (monocyte) subpopulation of cells, rather than the non-adherent lymphocytes (data not shown).

### Effect of increasing LBP on the LPS-dependent respiratory burst

To determine the signaling pathways involved in the respiratory burst, we first tested the effect of increasing concentrations of LBP. Whereas LPS-dependent IL-8 release by PBMC was LBP-independent (Figure 3A), superoxide production was significantly enhanced by LBP (Figure 4B, squares). Moreover, even at LBP concentrations comparable to those seen in the acute phase response (> 1 μg/ml), LBP was stimulatory, not inhibitory (data not shown). While increasing concentrations of LBP affected the magnitude of the response, they did not significantly alter the kinetics of the response, with maximal superoxide production occurring at ~15-20 minutes, and a return to near baseline levels by 1 h (data not shown).

### Effects of antibodies to TLR4 and CD14 on the LPS-dependent respiratory burst

**Figure 5:**
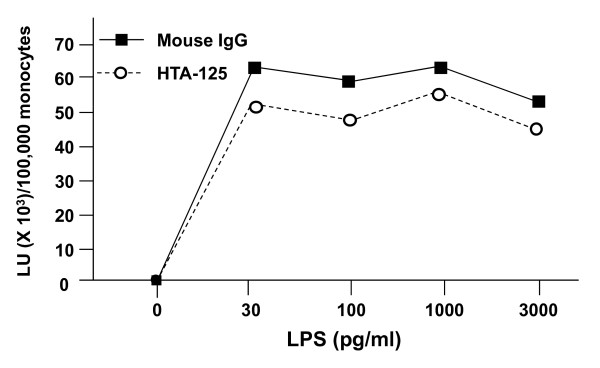
**Effect of anti-TLR4 blocking antibody on endotoxin-induced superoxide production by PBMC**. Human PBMC (~1.0 × 10^6 ^total cells/well, 100,000 monocytes) were prepared in 100 μl of incubation buffer containing 2 μg/well of mouse non-immune IgG or of HTA-125, and were placed in individual wells of a 96-well white Optiplate. Plates were incubated at 37°C for 30 min. Plates were then cooled on ice, and 100 μl of incubation buffer containing 0.2 mM lucigenin and 2× the final indicated concentration of endotoxin was added to the wells. The plates were then placed in the luminometer and two-sec readings were taken at 37°C every 2 min for 180 min. The resulting readings in relative light units (LU) were summed to provide a measure of activity. Data are expressed as total LU (in thousands) per 100,000 monocytes and represent the mean of duplicate samples. There were no significant differences between the mouse IgG concentration curve and the HTA-125 concentration curve.

As noted above, LPS-induced IL-8 release was blocked by antibodies to both CD14 and TLR4 (Figure 2). In contrast, the TLR4 blocking antibody HTA-125, at a concentration that produced a marked rightward shift in dose-dependent IL-8 release induced by endotoxin, had little impact on the magnitude (Figure 5) or the kinetics (data not shown) of endotoxin-induced superoxide production. The effect of MEM-18, a blocking antibody to CD14, was more variable. When used at a concentration that completely blocked endotoxin-induced IL-8 release, MEM-18 partially inhibited endotoxin-dependent superoxide production by monocytes from most donors, ranging from ≅ 10% to 100% inhibition, with an average of 55% (n = 4 donors, data not shown). The basis for this donor variability is unknown, but may reflect the relative abundance of membrane-bound CD14, donor-specific differences in antibody-CD14 interactions, and/or the presence of other membrane proteins that substitute as cofactors for endotoxin-dependent activation of the NADPH oxidase complex.

To test the possibility that members of the integrin family might contribute to endotoxin-induced superoxide release by monocytes, we performed a series of preliminary experiments in which we incubated PBMC with antibodies to CD11b/CD18 and CD11c/CD18 for 30 min before the addition of endotoxin. Neither antibody alone had an appreciable effect on endotoxin-induced superoxide production (data not shown); however, when both antibodies were used together, superoxide production was significantly reduced. Moreover, when both anti-integrin antibodies were combined with MEM-18, superoxide production was reduced to near-baseline levels in some donors, although this effect was not seen consistently.

### Effect of statins on endotoxin-dependent responses

**Figure 6:**
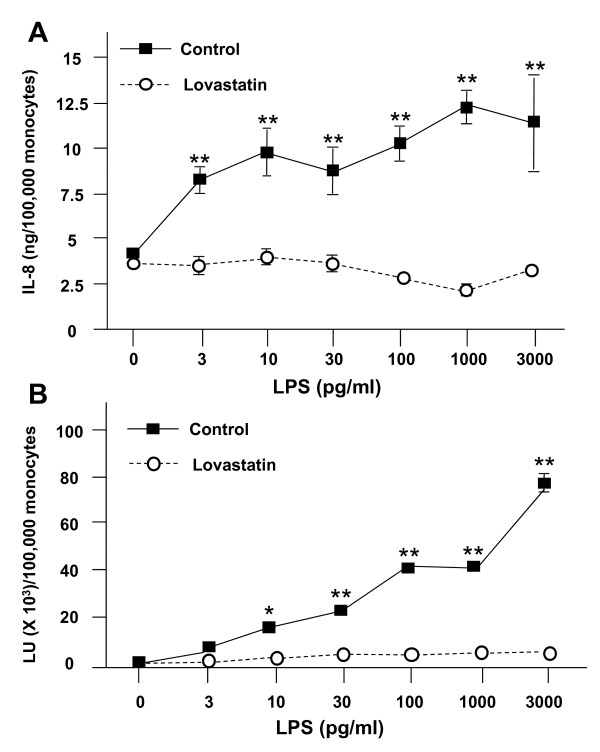
**Lovastatin inhibits endotoxin-induced IL-8 release and superoxide production**. Freshly isolated PBMC (~1.0 × 10^6 ^total cells/well, 100,000 monocytes) were prepared in 200 μl of incubation buffer with and without 1.5 μM lovastatin, and samples were incubated at 37°C for 16 h. (A) Cells were pelleted, washed gently, and resuspended in 200 μl of incubation buffer containing 100 ng/mL of LBP, and the indicated concentration of endotoxin. Cultures were then incubated at 37 °C for 6 h. At the end of the incubation period, cells were pelleted, and buffer samples were frozen at -20°C for subsequent IL-8 analysis by ELISA. Data are expressed as the mean ± S.E.M. of triplicate samples. (B) Cells were pelleted, washed gently, and resuspended on ice in individual wells of a 96-well white Optiplate in 200 μl of HBSS/0.1% HSA/0.1% G containing 100 ng/mL of LBP, 0.1 mM lucigenin, and the indicated concentration of endotoxin. The plates were then placed in the luminometer and two-sec readings were taken at 37°C every 2 min for 180 min. The resulting readings in relative light units (LU) were summed to provide a measure of activity. Data are expressed as total LU (in thousands) per 100,000 monocytes and represent the mean ± S.E.M. of 4 replicate samples; error bars for some conditions were so small they are obscured by the symbols. (A) **p < 0.001 for the no lovastatin control relative to the lovastatin value at the same LPS concentration. (B) *p < 0.05, **p < 0.001 for the no lovastatin control relative to the lovastatin value at the same LPS concentration. Similar results to A and B were seen in 3 independent experiments.

In addition to their lipid-lowering properties, statins have recently been shown to induce anti-inflammatory effects in a number of model systems including those using human monocytes[22-24]. In particular, their ability to modulate TLR-4 expression is being investigated in the treatment of sepsis and septic shock[25]. Moreover, pertinent to vascular biology, our laboratory recently showed that statins inhibit LPS-induced pro-inflammatory signaling in human blood vessels and in human coronary artery endothelial and smooth muscle cells, and that this effect is due to inhibition of geranylgeranylation of one or more proteins in the proximal part of the endotoxin signaling pathway [26,27]. Thus, we tested whether statins could inhibit LPS-dependent pro-inflammatory responses in monocytes. Figure 6 shows that both IL-8 release (Figure 6A) and superoxide production (Figure 6B) are inhibited by 1.5 μM of lovastatin.

## Discussion

Taken together, our results suggest that two important pro-inflammatory responses to low level endotoxin, IL-8 release and superoxide production, are activated by two distinct signaling pathways in monocytes freshly isolated from healthy human donors (Table 1). One is the rapid (minutes) endotoxin mediated oxidative burst leading to production of the reactive oxygen species superoxide anion by the NADPH oxidase complex. The other is release of the pro-inflammatory cytokine IL-8, involving activation of multiple transcription factors including NF-κB. We found that the endotoxin-mediated respiratory burst is LBP-dependent yet an anti-TLR4 antibody had no effect on the endotoxin-mediated respiratory burst as measured here. As inhibition by the CD14 blocking antibody was somewhat variable, there may a component of the activation pathway that is CD14 independent. Contrastingly, in our experimental conditions IL-8 release appears to be LBP-independent, CD14- and TLR4-dependent.

**Table 1:** Endotoxin-dependent IL-8 release and ROS formation by freshly isolated human monocytes is regulated by distinct signaling pathways.

IL-8 Release	ROS Production
LBP-independent	LBP-dependent
CD14-dependent	Partially CD14-dependent
TLR4-dependent	TLR4-independent
Inhibited by lovastatin	Inhibited by lovastatin

There is ample evidence for multiple endotoxin signaling pathways. Best characterized is the pathway in which LBP facilitates the transfer of endotoxin monomers to CD14, which in turn presents the endotoxin monomer to the MD-2/TLR4 complex [28-32]. The transmembrane domain of TLR4 then transmits the signal *via *a series of adapter proteins and kinases (*e.g*., MyD88, IRAK, TRAF6, NIK, IκB, and NFκB) to the interior of the cell and ultimately to the nucleus, resulting in gene expression. However, a number of reports in recent years have suggested the existence of alternate signaling pathways[33].

Studies using LBP knockout mice are consistent with a physiologically relevant LBP-independent inflammatory response. While whole blood from these animals was 1000-fold less responsive to endotoxin *in vitro *studies, nevertheless when the mice were injected with LPS, no significant differences between wild-type and LBP knockout mice (as measured by TNF release) were observed [34]. In addition, responses to LPS can also be affected by LBP concentration and the presence or absence of other serum proteins[20,35-38]. Thompson *et al*. demonstrated in THP-1 cells that adding high-density lipoprotein (HDL) augmented the response to LPS in otherwise inhibitory LBP concentration conditions. Interactions may also take place between TLR4 and HMGB1 as indicated by Youn *et al*.[39]. In our specific set of experimental circumstances, we found that LPS-induced IL-8 release by monocytes was LBP-independent (Table 1), although in repeated experiments, this finding was in contrast to results with differentiated macrophages from the same donor. This might be explained by the limiting stoichiometry and molar relationships between relative amounts of endotoxin, LBP, CD14 and TLR4[40]. For instance, as the concentration of LPS increases, the ratio of LBP to LPS decreases, and activation increases. It is possible that in our monocyte isolation, inadequate CD14 or TLR4 expression may have limited cellular activation and IL-8 release. Furthermore, this would also explain why results from differentiated macrophages, with likely a greater expression of CD14, contrasted with the previous results in monocytes from the same donor. We also observed decreasing LPS-induced IL-8 release with decreasing albumin concentrations (data not shown), suggesting that the human serum albumin in our incubation buffer (and in plasma) was facilitating endotoxin-binding and delivery in our experimental model[21]. It is possible from what is known from our studies as well as others that serum proteins may variably contribute or inhibit LPS monomer delivery and hence LPS stimulatory activity, depending on the experimental and/or physiologic conditions.

To determine whether CD14 mediated LPS-induced IL-8 release or the respiratory burst in freshly isolated PBMC, we performed studies with the anti-CD14 blocking antibody MEM-18, which binds to an epitope on the endotoxin-binding site of CD14. MEM-18 completely inhibited IL-8 release; however, the ability of the antibody to block the respiratory burst was highly variable (ranging from approximately 10% to 100%) and appeared to be specific to individual donors (Table 1). Other investigators have reported evidence of CD14-independent signaling, particularly with relatively high (> 100 ng/ml) concentrations of endotoxin [8-10]. Human and murine responses to LPS differ in that the concentrations of endotoxin challenge necessary to elicit responses in mice are much greater[41]. For instance, Maitra et al. demonstrated the contribution of IRAK-1, a known activator of NOX-1 enzymatic activity, in the rapid generation of ROS induced by LPS (100 ng/mL) in murine cells[11]. Perera *et al*. found a CD14-independent signaling pathway in macrophages from CD14-knockout mice [42]. In this model system, cytokine release in response to low doses of endotoxin (1 or 10 ng/ml) showed an absolute requirement for CD14 (either the membrane-bound or soluble form, as found in serum); however, at concentrations of 1 μg/ml or higher, endotoxin induced equal or greater production of TNF-α and IL-1β in CD14-knockout as compared to wild-type mice. Kimura *et al*. also found evidence of a CD14-independent endotoxin signaling pathway in a murine B cell line [43]. In these experiments, IL-6 mRNA expression as well as ^3^H-thymidine incorporation as a measure of cellular proliferation were increased upon stimulation with endotoxin at 1 μg/ml even in the presence of anti-murine CD14 monoclonal antibody.

The potential complexity of endotoxin signaling is suggested by the model of a CD14 receptor complex proposed by Triantafilou *et al*. [8,44,45]. Using fluorescence recovery after photobleaching method (FRAP), these authors showed that endotoxin initially binds to CD14, a GPI-anchored receptor, before being transferred to other receptors such as MD-2. The MD-2:LPS complex associates with the TLR4 ectodomain for signal transduction[5,32]. Interestingly, there is evidence to suggest that additional receptors may play a role in the transmembrane signal transduction. These include CD11/CD18 integrins, the CD55 receptor, and a receptor cluster consisting of four different molecules (Heat shock proteins 70 and 90, chemokine receptor 4, and growth differentiation factor 5).

One possible mechanism for the partially CD14-independent signaling we observed is through the leukocyte integrin transmembrane receptor CD11/CD18 family [46-49]. There are three subtypes of this receptor. CD11a/CD18 participates in leukocyte-endothelial cell interaction and is found in all white cells. CD11b/CD18 is found mainly on monocytes and neutrophils and functions as complement receptor for C3b. The activity of this subgroup of receptors is enhanced in human PMN when exposed to endotoxin in a CD14-dependent manner [50]. The CD11c/CD18 complex, though function is unclear, is found in many cells including monocytes and may be the main alternative to CD14 involved in the identification of endotoxin [51]. CHO cells transfected with CD11c/CD18 were able to recognize and respond to endotoxin independent of CD14 even at low concentration in the range of 1 ng/ml [46]. However this is not a necessary condition since cells with mCD14 can release pro-inflammatory cytokines when exposed to endotoxin in the absence of CD18 receptors [52]. To further support a potential contribution of CD11/CD18 receptors to CD14-independent signaling, we found decreased superoxide production in response to PBMC exposure to endotoxin only with the combined use of CD11b/CD18 and CD11c/CD18 antibodies, but not when used individually. Further studies are warranted to elucidate the contributory mechanisms of specific serum proteins and receptor subtypes in CD14-dependent and independent endotoxin signaling.

## Conclusions

The endotoxin-CD14 complex initiates signaling by engaging TLR4 and the co-receptor MD-2 [5,32,53]. Cellular responses to endotoxin are partly determined by concentration and type of LPS (rough or smooth), length of exposure, as well as the affected cell type. Together these factors determine cell surface receptor expression, cytokine production, and ROS generation [54,55]. Recent evidence suggests that TLR4 plays an important role in atherogenesis [56]. Specifically, the Asp299Gly polymorphism, which attenuates endotoxin signaling, is associated with a decreased risk of atherosclerosis [57]. TLR4 function has been widely studied in monocytes and macrophages and was recently examined in human blood vessels [27] and human coronary artery endothelial and smooth muscle cells [3] by our laboratory. Endotoxin stimulates ROS production in non-immune cells through a direct interaction between TLR4 and NOX4 [58,59]. However, we found that while LPS-induced IL-8 release by human monocytes was TLR4-dependent (*i.e*., blocked by HTA-125), superoxide formation was largely TLR4-independent (Table 1). This suggests that activation of the NADPH oxidase by LPS is mediated through alternative receptors in human monocytes. A potential candidate pathway includes the MAC1 receptor, which is expressed by monocytes [60] and mediates endotoxin-dependent ROS formation in microglial cells [61]. Table 1 also summarizes the effects of lovastatin treatment. Our studies with lovastatin provide additional evidence for the anti-inflammatory effects of these compounds. Given the important role that monocyte activation is believed to play in acute coronary syndromes, our findings may also help to explain the significant reductions in coronary events observed in patients taking statins.

Taken together, our results suggest that two important pro-inflammatory responses to low concentrations of endotoxin, IL-8 release and superoxide production, are activated by two distinct signaling pathways in human PBMC. To our knowledge, this is the first study that evaluates the presence of alternative signaling pathways in freshly isolated human PBMC from healthy donors that are exposed to clinically relevant endotoxin levels.

## Competing interests

The authors declare that they have no competing interests.

## Authors' contributions

All contributors to this manuscript have made substantive intellectual contributions to this study. Each author has participated sufficiently in this work to take public responsibility for appropriate portions of the content. Each author has reviewed and given approval for this version to be published.

Specifically, AB prepared the manuscript for submission and participated in the analysis and interpretation of the data. LS conceived of the study, performed necessary assays, and wrote the first draft of the manuscript. WS coordinated the sample acquisition from human volunteers and drafting of the manuscript. SM carried out the assays and analyzed the data. ED analyzed data and helped draft the manuscript. NW participated in the design, coordination of the study and helped draft the manuscript. GD participated in the design of some experiments, analyzed data, prepared figures and assisted in the first draft of the manuscript.
